# Barrett’s oESophagus trial 3 (BEST3): study protocol for a randomised controlled trial comparing the Cytosponge-TFF3 test with usual care to facilitate the diagnosis of oesophageal pre-cancer in primary care patients with chronic acid reflux

**DOI:** 10.1186/s12885-018-4664-3

**Published:** 2018-08-03

**Authors:** Judith Offman, Beth Muldrew, Maria O’Donovan, Irene Debiram-Beecham, Francesca Pesola, Irene Kaimi, Samuel G. Smith, Ashley Wilson, Zohrah Khan, Pierre Lao-Sirieix, Benoit Aigret, Fiona M. Walter, Greg Rubin, Steve Morris, Christopher Jackson, Peter Sasieni, Rebecca C. Fitzgerald, on behalf of the BEST3 Trial team

**Affiliations:** 1 School of Cancer and Pharmaceutical Sciences, Faculty of Life Sciences & Medicine, King’s College London, London, UK; 2 Cancer Prevention Trials Unit, Centre for Cancer Prevention, Wolfson Institute of Preventive Medicine, Queen Mary University of London, London, UK; 3 Department of Histopathology, Addenbrooke’s Hospital, Cambridge, UK; 4 MRC Cancer Unit, Hutchison/MRC Research Centre, University of Cambridge, Cambridge, UK; 5 Leeds Institute of Health Sciences, University of Leeds, Leeds, UK; 6 Astra Zeneca, Cambridge, UK; 7 The Primary Care Unit, Department of Public Health and Primary Care, University of Cambridge, Cambridge, UK; 8 Institute of Health and Society, Sir James Spence Institute, Royal Victoria Infirmary, Newcastle University, Newcastle upon Tyne, UK; 9 Department of Applied Health Research, University College London, London, UK; 10 MRC Biostatistic Unit, University of Cambridge, Cambridge, UK

**Keywords:** Heartburn, Acid reflux, Early detection, Oesophageal cancer, Biomarker, Endoscopy, Cost effectiveness, Acceptability, Quality of life, Medical device

## Abstract

**Background:**

Early detection of oesophageal cancer improves outcomes; however, the optimal strategy for identifying patients at increased risk from the pre-cancerous lesion Barrett’s oesophagus (BE) is not clear. The Cytosponge, a novel non-endoscopic sponge device, combined with the biomarker Trefoil Factor 3 (TFF3) has been tested in four clinical studies. It was found to be safe, accurate and acceptable to patients.

The aim of the BEST3 trial is to evaluate if the offer of a Cytosponge-TFF3 test in primary care for patients on long term acid suppressants leads to an increase in the number of patients diagnosed with BE.

**Methods:**

The BEST3 trial is a pragmatic multi-site cluster-randomised controlled trial set in primary care in England. Approximately 120 practices will be randomised 1:1 to either the intervention arm, invitation to a Cytosponge-TFF3 test, or the control arm usual care. Inclusion criteria are men and women aged 50 or over with records of at least 6 months of prescriptions for acid-suppressants in the last year. Patients in the intervention arm will receive an invitation to have a Cytosponge-TFF3 test in their general practice. Patients with a positive TFF3 test will receive an invitation for an upper gastro-intestinal endoscopy at their local hospital-based endoscopy clinic to test for BE.

The primary objective is to compare histologically confirmed BE diagnosis between the intervention and control arms to determine whether the offer of the Cytosponge-TFF3 test in primary care results in an increase in BE diagnosis within 12 months of study entry.

**Discussion:**

The BEST3 trial is a well-powered pragmatic trial testing the use of the Cytosponge-TFF3 test in the same population that we envisage it being used in clinical practice. The data generated from this trial will enable NICE and other clinical bodies to decide whether this test is suitable for routine clinical use.

**Trial registration:**

This trial was prospectively registered with the ISRCTN Registry on 19/01/2017, trial number ISRCTN68382401.

**Electronic supplementary material:**

The online version of this article (10.1186/s12885-018-4664-3) contains supplementary material, which is available to authorized users.

## Background

Incidence of oesophageal adenocarcinoma (EAC) has increased six-fold since the 1970s and carries a dismal prognosis (13% 5-year survival) despite advances in neo-adjuvant therapy and surgery [1]. Clinical guidelines have focused on urgent referral for those with alarm symptoms, specifically dysphagia, chronic gastro-intestinal bleeding, weight loss, persistent vomiting, anaemia or an epigastric mass [2]. Routine referral is advised for those with reflux symptoms that persist despite recommended lifestyle and pharmacological management strategies [3]. General Practice (GP) referral rates vary widely and low endoscopy referral rates have been linked with poor outcomes from oesophageal cancer [4].

Approximately 3 to 6% of individuals with reflux predominant symptoms may have Barrett’s oEsophagus (BE), the precursor lesion to EAC. However, only 20 to 25% of patients with BE are diagnosed [5]. It is estimated that the burden of EAC could be reduced by up to 50% as a result of increasing the proportion of individuals with reflux predominant symptoms who are investigated [6]. This is a formidable task since dyspepsia and gastro-oesophageal reflux disease (GERD) affect between 5 and 20% of the population [7] and account for up to 10% of GP consultations in the UK.

Endoscopic treatment of BE, which progresses through dysplastic and superficially invasive stages, can prevent the development of EAC [8]. Indeed, endoscopic treatment is now recommended for patients with low and high grade dysplasia following new randomised controlled trial evidence [9, 10]. By identifying and referring to endoscopy those most likely to have BE, it should be possible to reduce mortality from EAC in patients with reflux who would not otherwise receive endoscopy.

### Cytosponge diagnostic test for Barrett’s oESophagus

**Fig. 1:**
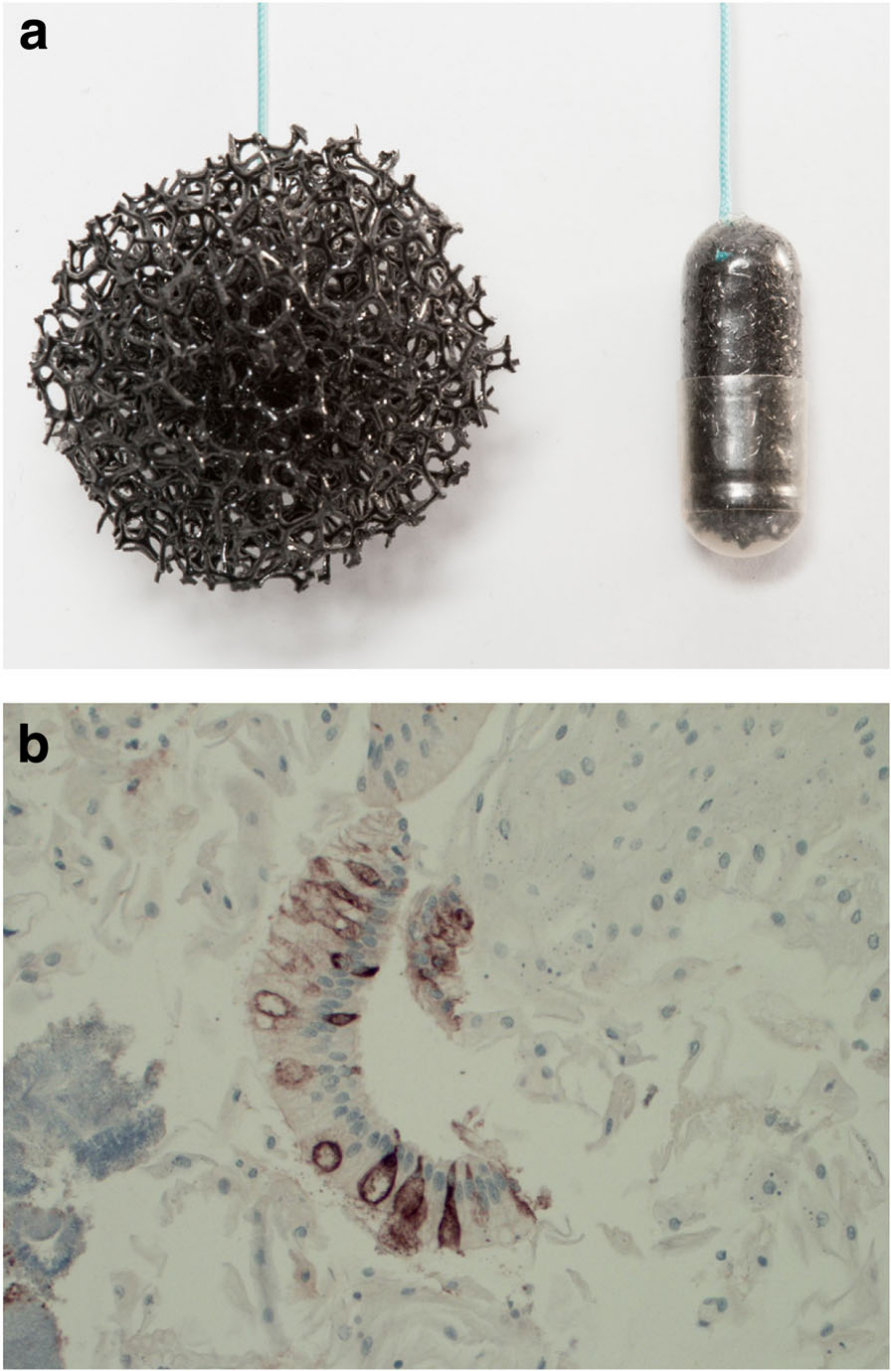
(**a**) Cytosponge™ expanded (left) and in gelatin capsule (right) (**b**) representative picture of positive TFF3 staining in a sample from a patient with BE (× 20 magnification)

A non-endoscopic diagnostic modality for BE has been developed which involves a device called the Cytosponge™ combined with molecular biomarker Trefoil Factor 3 (TFF3) [11] (Fig. 1).

The Cytosponge™ consists of a Class I, non-CE marked 3 cm diameter, polyester, medical grade mesh sphere on a string, compressed within a gelatine capsule. The capsule is swallowed while holding onto the string. After 5 min, the gelatine capsule has dissolved allowing the sphere to expand. Using the string the sphere is pulled from the stomach to the oesophagus and mouth thus collecting cells from the whole of the BE segment [12], as well as from the squamous oesophagus and oropharynx. The sample is put into a preservative, processed, and assessed for the presence of BE via immunohistochemical staining for TFF3.

A series of four clinical studies have been carried out so far with the following findings (see Table 1). First, no serious adverse events were attributed to the Cytosponge™ after administering this test over 2000 patients, showing that it is a safe intervention [11, 13–16]. Second, the Cytosponge™ is acceptable to patients with a mean score of 6.0 (95%CI 5.0–8.0) on a visual analogue scale from zero (worst experience) to 10 (best experience) reported by patients [11, 13–16]. Third, the Cytosponge intervention was shown to be transferable to the NHS: 27 nurses were trained with a single training session in 11 sites and sample processing was run in an NHS pathology laboratory [13, 17]. Fourth, a feasibility study conducted in 504 patients in 11 general practices showed that the intervention can be applied to primary care [13]. Fifth, the Cytosponge™-TFF3 test was found to be accurate for diagnosing BE regardless of patient cohort or study setting. Last, this test is cost-effective in comparison to the usual care. A micro-simulation model suggested a gain of 0.015 QALYs (Quality adjusted life years) and an incremental cost effectiveness ratio (ICER) of $15,700 per QALY for Cytosponge™ versus endoscopic diagnosis of BE followed by endoscopic treatment [18].

**Table 1:** Studies summary, sensitivity and specificity of the Cytosponge™-TFF3 test per segment length

Study Ref n#	Publication Year	Study type	Setting	BE length	Sensitivity % (95% CI)	Specificity % (95% CI)
Pilot [14]	2008	Cohort	2^ary^ care	≥C1	78.0 (64.0–89.0)	94.0 (87.0–98.0)
BEST1 [12]	2010	Prospective	1^ary^ care	≥C1	73.3 (44.9–92.2)	93.8 (91.3–95.8)
				≥C2	90.0 (55.5–99.7)	93.5 (90.9–95.5)
BEST2 [13]	2014	Case:control	2^ary^ care	≥C1	79.5 (75.9–82.9)	92.4 (89.5–94.7)
				≥C2	83.9 (80.0–87.3)	
				≥C3	87.2 (83.0–90.6)	
CASE1 [15]	2015	Cohort	2^ary^ care	≥C1 or ≥ M3 ≥C3	95.4 (86.9–98.9) 96.8 (83.7–99.5)	N/A

### Rationale

Our long-term vision is for the Cytosponge™-TFF3 technology to be adopted as a triage test within the standard primary care clinical pathway for patients on treatment with acid suppressants (proton pump inhibitors (PPIs) or H2 receptor antagonists (H2RAs)) who do not fulfil the referral criteria for endoscopy. This strategy will increase the proportion of patients diagnosed with BE, and in turn allow for endoscopic therapy and monitoring for those at greatest risk of EAC.

### Objectives

Primary and secondary objectives, and endpoints are summarised in Table 2 and described below.

**Table 2:** BEST3 Trial objectives and endpoints

Objective	Endpoint	Usual care arm	Intervention arm
Primary objectives			
1. To compare histologically confirmed BE diagnosis between intervention and control	BE diagnosis within 12 months of joining the study (excluding BE found on random 12 month research endoscopy that will occur following 12 month snapshot).	Anonymised data aggregated by sex and age group from: - GP databases - Confirmed by upper GI endoscopy (biopsy result) as recorded in the GP record within 12 months	Anonymised data aggregated by sex and age group from: - GP databases - Confirmed by upper GI endoscopy (biopsy result) as recorded in the GP record within 12 months In addition, for patients with Cytosponge™ -TFF3 test: - Endoscopy record and pathology results
Secondary objectives			
(i) To evaluate the cost of the Cytosponge™-TFF3 test versus usual care Objective (ii) To evaluate the cost-effectiveness of the Cytosponge™-TFF3 test versus usual care	(i) Mean cost per patient receiving the Cytosponge™-TFF3 test versus usual care. Costs to include costs of diagnosis using the Cytosponge™-TFF3 test, endoscopies and biopsies, endotherapy, oesophagectomy, medications, and follow-up in primary and secondary care. (ii) Incremental cost per QALY gained of the Cytosponge™-TFF3 test versus usual care	(i) Volume of resource use (endoscopies and biopsies, endotherapy, oesophagectomy, medications, and follow-up in primary and secondary care) from patient records. Unit costs (of each item of resource use) from published sources. (ii) Calculation of incremental cost per QALY gained to be based on a pre-existing model, supplemented with new data from the Trial.	(i) Volume of resource use (Cytosponges™, endoscopies and biopsies, endotherapy, oesophagectomy, medications, and follow-up in primary and secondary care) from patient records. Unit costs (of each item of resource use) from published sources. (ii) Calculation of incremental cost per QALY gained to be based on a pre-existing model, supplemented with new data from the trial.
To assess the diagnostic accuracy of the Cytosponge™ in primary care	Positive Predictive Value (PPV), Negative Predictive Value (NPV) in relation to the length of BE	N/A	- PPV: proportion of TFF3 positive results confirmed to have BE by endoscopy - NPV: proportion of TFF3 negative cases confirmed to not have BE by endoscopy (10% endoscopy)
To assess diagnostic performance of Cytosponge™ in detecting severity for BE	Score of BE severity based on BE biopsy results	N/A	Endoscopy reports for participants
To report on the sampling adequacy	Inadequacy rate (same as BEST1 and BEST2)	N/A	CRF to capture: - Sample sufficient to generate result - Proportion of Cytosponge™ samples with < 5 and < 1 columnar cells (minimal standard)
To confirm the endoscopy referral rate in the intervention arm	Proportion positive out of all adequate TFF3 tests and out of all patients swallowing a Cytosponge™ at least once	N/A	Cytosponge™-TFF3 test results
To report on patient acceptability for Cytosponge™	(i) Willingness: proportion of patients offered Cytosponge™ test who accept (ii) Cytosponge™ swallowing failures (iii) Increased and decreased cancer worry due to procedure and results (iv) Long term emotional or physical harm caused by procedure (v) Test experience (vi) Willingness to have repeat procedure	N/A	(i) Number of patients invited vs those consenting to Cytosponge™-TFF3 test (ii) Number of patients who fail to swallow and number of attempts Acceptability measures at baseline: (iii) STAI-6 (iv) Perceived risk of oesophageal cancer Acceptability measures at day 7–14: (iii) Perceived risk of oesophageal cancer (iv) STAI-6 (v) A visual analogue scale to rate experience (v-vi) the Inventory to Assess Patient Satisfaction, (iii – v) up to 30 qualitative patient interviews
To assess physician/nurse acceptability of the Cytosponge™	Experience and acceptability of Cytosponge™: administration, skills, reliability, side effects, user information	N/A	Qualitative interviews of clinical staff
To report on the safety of the Cytosponge™ in primary care	Any ADE/ARs reported by patients up to 7 days post swallowing	N/A	Contact card given in case of ADE/SADE and 7-day telephone call
(i) To understand how much BE is missed in current management of patients (ii) To compare undiagnosed BE in general population vs those who have been tested with Cytosponge™-TFF3	- BE at 12 months - PPV for endoscopy referral, i.e. Cytosponge™ vs current GP criteria for referring for an endoscopy to look for BE	12 month endoscopy for 10% of patients not requiring a clinically indicated endoscopy in time period of the study	Confirmatory endoscopy for patients with positive result and endoscopy findings from patients with negative result who accept research endoscopy at 12 months.
To assess prevalence of benign oesophageal conditions	Prevalence of oesophageal conditions aside from BE in primary care population consulting with reflux symptoms	Endoscopy findings in 10% patients endoscoped	Endoscopy findings in 10% patients endoscoped and on Cytosponge™ test (via pathology assessment)
Epidemiology: (i + ii) To confirm the prevalence (and incidence) of BE in both arms (iii) To confirm the prevalence (and incidence) of OC diagnosis (by stage) in both arms (iv) To confirm the prevalence (and incidence) diagnosis cancers of the gastric cardia (by stage) in both arms (v) To produce a model to predict the reduction in EAC related mortality from this strategy	(i) Diagnosis of BE (ii) Diagnosis BE with dysplasia (iii) Diagnosis of oesophageal cancer (OC) + stage at diagnosis (iv) Diagnosis of cancer of the gastric cardia + stage at diagnosis (v) Percentage of expected reduction in EAC mortality based on prevalence of BE if Cytosponge™ test introduced	(i-iv) Aggregate data from GP databases	(i-iv) Aggregate data from GP databases (i-iv) Endoscopy data from 10% endoscopy offered at 12 months (i-iv) Cytosponge™ patients: - Cytosponge™ findings - Endoscopy findings
10% endoscopy invitation across both arms: (i) Acceptability of endoscopy (ii) Perceptions around Cytosponge™ use and reliability (iii) Number of BE diagnoses 12 months after negative TFF3 tests	(i) Comparisons between acceptance of invitation to endoscopy compared to Cytosponge™ test (ii) Proportion of patients with Cytosponge™ test who take up invitation to endoscopy (iii) Number of BE diagnosis in TFF3 negative patients in intervention arm	(i) 10% endoscopy: uptake of invitation to endoscopy	(i) 10% endoscopy: uptake of invitation to endoscopy for all participants who have not received the CytospongeP™ P (excluding ineligible patient and non-attendees for Cytosponge™) (i) CytospongeP™ Ptest invitation uptake (ii) Endoscopy uptake amongst patients with previous Cytosponge™ test (iii) BE diagnosis amongst patients with previous Cytosponge™ test
Objective			
Longer-term objectives			
Epidemiology: For up to 10 years, to confirm the prevalence (and incidence): (i + ii) of BE in both arms (iii) of OC diagnosis (by stage) in both arms (iv) of cancers of the gastric cardia (by stage) in both arms (iv) To undertake modelling to predict the reduction in EAC related mortality from this strategy	(i) Diagnosis of BE (ii) Diagnosis of BE with dysplasia (iii) Diagnosis of OC + stage at diagnosis (iv) Diagnosis of cancer of the gastric cardia + stage at diagnosis (v) Percentage of expected reduction in EAC mortality based on prevalence of BE if Cytosponge™ test introduced	(i-iv) Anonymised data from cancer registry flagging- conducted anonymously via novel encryption method (v) Based on BE prevalence, prevalence of BE with dysplasia, flagging with the cancer registry, ONS and HES datasets	(i-iv) Anonymised data from cancer registry flagging- conducted anonymously via novel encryption method (v) Based on BE prevalence, prevalence of BE with dysplasia, flagging with the cancer registry, ONS and HES datasets
Research and Development (including in future studies)	Genetic and biochemical risk factors for disease progression (germline and somatic variants and other biomarkers) including targeted, exome level and whole genome sequencing.	10% patients who have endoscopy- surplus material from biopsies	- Surplus Cytosponge™ material - Saliva samples (for TFF3 positive patients only) - Surplus endoscopy biopsies

#### Primary objectives

To compare histologically confirmed BE diagnosis between intervention and the control arms to determine whether the offer of the Cytosponge™-TFF3 test in primary care results in an increase in BE diagnosis within 12 months of study entry.

#### Secondary objectives

The main secondary objectives are to evaluate the cost and cost-effectiveness of the Cytosponge™-TFF3 test versus usual care. Other secondary objectives include confirming the safety and diagnostic accuracy of the Cytosponge™-TFF3 test and determining the uptake in primary care. Inviting 10% of BEST3 participants for a research endoscopy will enable us to assess the diagnostic accuracy of the Cytosponge™-TFF3 test in primary care. We will also assess the acceptability of the Cytosponge™-TFF3 test to patients, GPs and practice nurses, and their experiences of its use in primary care. Long term objectives are to confirm the prevalence and incidence of BE, EAC and cancer of the gastric cardia in a primary care population consulting with reflux predominant symptoms and to undertake modelling to predict the reduction in EAC related mortality from this strategy.

## Methods

### Design

**Fig. 2:**
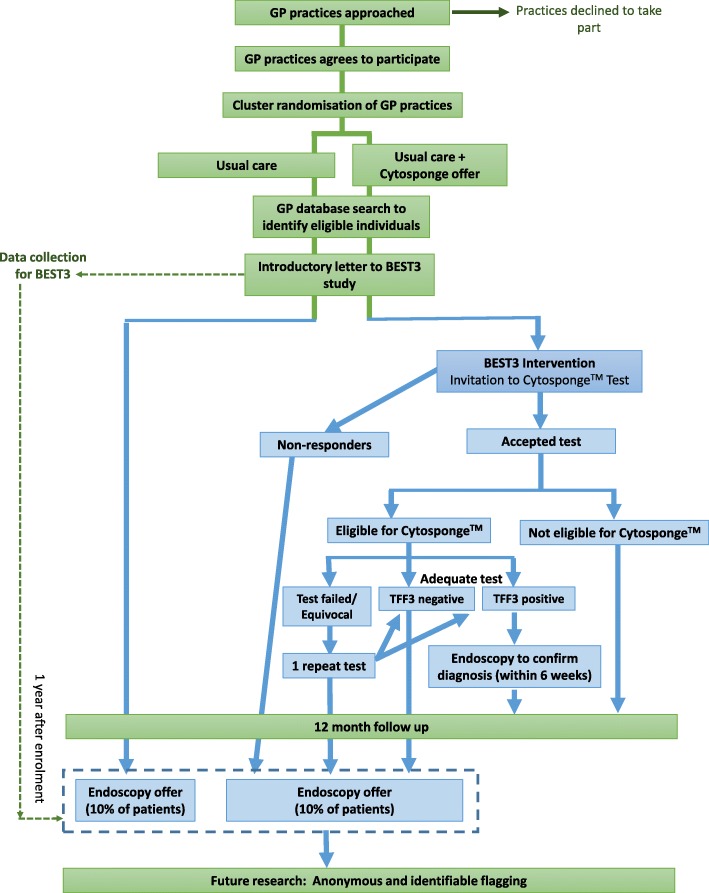
BEST3 trial design overview showing both BEST3 anonymous data collection steps (green) and intervention, Cytosponge™-TFF3 test or upper GI endoscopy related procedures (blue)

This is a pragmatic multi-site cluster randomised controlled trial where approximately 120 general practices will be randomised 1:1 to either the intervention or control arm (Fig. 2). Anonymised data will be collected from eligible patients in both arms at baseline and 1 year post entry into the study. Patients will be informed about being entered into BEST3 data collection by letter. Patients in the intervention arm will receive an invitation for a Cytosponge™-TFF3 test in their general practice. Patients with a positive TFF3 test will receive an invitation for an upper gastro-intestinal (GI) endoscopy at their local hospital-based endoscopy clinic to test for BE. In addition to the TFF3-positive patients, 10% of the patients in each arm (who have not had an endoscopy since the start of the trial) will be randomly selected to be invited for an endoscopy at approximately 12 months.

### Study setting

120 (but up to 150 practices as the project requires) will be recruited from six to seven clinical research networks (CRNs): Eastern, North Thames, North East and North Cumbria, Yorkshire and Humber, but other regions may participate as required. For Information Technology reasons, only practices using Egton Medical Information Systems (EMIS) or TTP’s SystmOne, the two most common systems, will be included in the study.

### Participants

Patients at participating general practices will be selected by GP staff. Inclusion and exclusion criteria for the different stages of BEST3 are outlined in Table 3. In brief, male and female patients aged 50 and over with records of at least 6 months of prescription for acid-suppressant medication (PPI or H2RA) in the last year will be eligible for BEST3 data collection. Patients who also have records of prescriptions for non-steroidal anti-inflammatory drugs (NSAIDs) or an upper GI endoscopy in the previous 5 years will be excluded. These eligibility criteria are based on GP database records, which are not always complete. However, as they are used to increase the power of the trial and not for safety reasons, they only have to be applied based on data as it is available.

**Table 3:** Inclusion and Exclusion criteria for the BEST3 trial

Inclusion criteria	Exclusion criteria for		
	BEST3 data collection	Cytosponge procedure	Upper GI endoscopy
• Male and female • Aged ≥50 • Records of ≥6 months of prescription for acid-suppressant medication in the last year	• Recorded regular prescriptions of NSAIDs • Recorded upper GI endoscopy in the previous 5 years as identified from the practice database • Recorded diagnosis of a current or previous oro-pharynx, oesophageal or gastro-oesophageal tumour • Recorded diagnosis of BE • Unable to attend the GP surgery • Deemed not fit enough by their GP, including lacking capacity	• Meeting the guidelines for an urgent endoscopy referral according to NICE guidelines • Recorded diagnosis of an oro-pharynx, oesophageal or gastro-oesophageal tumour (T2 staging and above), or symptoms of dysphagia • Difficulty in swallowing due to a known cerebrovascular accident or neurological disorder • Recorded oesophageal varices, cirrhosis of the liver • Inability to temporarily discontinue anti-thrombotic medication prior to procedure • Having eaten and drank within the preceding 4 h • Received prior surgical intervention to the oesophagus • Known pregnancy • Lacking capacity to provide informed consent	• Upper GI endoscopy during the study period • Severe hypertension (e.g. systolic > 200 diastolic > 100) • Myocardial infarction or any cardiac event within the previous 6 months • Cerebrovascular event or other neurological disorder where swallowing has been affected within the previous 6 months • Any previous treatment such as Photodynamic therapy (PDT) or Radio Frequency Ablation (RFA) to the oesophagus • Anticoagulation therapy/ medication on day of procedure (warfarin, heparin or tinzaparin) according to local guidelines • Other medical condition: low platelets or blood abnormalities that may cause excessive bleeding post procedure • Eaten or drank within the previous 6 h • Preference for sedation and has not brought anyone to accompany them at home. Follow local guidelines • Known pregnancy • Lacking capacity to provide informed consent

#### Number of participants

A minimum of 9000 patients will be entered into the BEST3 trial.

### Randomisation

All patients aged 50 and over who have received at least 6 months’ supply of an acid suppressant drug (either PPI or H2RA) in the last year will be identified by carrying out a database search on coded clinical information. If a practice database search identifies more than 100 eligible patients, 100 patients will be randomly selected to be included in the trial. Once the practice consents to participate in BEST3, each practice will be randomised via block randomisation and stratified by number of eligible patients. Three groups of approximately 40 practices are proposed, each with the following number of patients:

- Stratum 1 (small practices): 50–60
- Stratum 2 (medium practices): 61–74
- Stratum 3 (large practices): 75–100.

The number of patients invited in each stratum and/or the number of strata used might be increased depending on uptake of the Cytosponge. Once a practice has consented to participate in the trial, a database search will be carried out to determine the number of eligible patients and as part of which stratum they will be randomised. Practices will be randomised 1:1 between the intervention and control arms. Randomisation will be balanced by geographical area to ensure that Cytosponge™ clinics will have similar number of bookings in each area. However, as the number of eligible patients in the different practices who will participate is not definite, the number of practices per stratum and the number of strata overall will be adjusted as necessary. The process will be managed so that number of practices in each arm and each stratum will be equal and that the numbers of participants per practice within a stratum will be similar.

### Dates and duration of the trial

Start date: September 2016.

Total duration: 36 months.

### Consent procedures

Patients will be entered into different stages of the trial at different levels of consent.

#### Consent at practice level (opt in)

Firstly, consent will occur at practice level. GPs will provide consent that the practice can be randomised and participants contacted within the BEST3 Trial. GPs will furthermore consent to aggregate anonymised patient data being collated from their practice database and patient notes.

#### Introductory letter provided to patients about use of anonymous data (BEST3 data collection)

All participants across both arms will receive an information letter from their practice outlining that anonymous data collected in the course of their routine care will be included in the study. They will have 14 days to opt out of having their anonymised data collected and analysed as part of BEST3.

#### Written consent for BEST3 intervention and endoscopy (opt in written consent)

Written (individual-level) consent will be obtained before carrying out any procedures. All participants receiving a Cytosponge™–TFF3 test or endoscopy as part of the study will be individually-consented to have this procedure and for the associated clinical data to be collected.

### BEST3 introductory letter and anonymous data collection

#### BEST3 introductory letter

Eligible patients to be included in the study will be sent the introductory letter about the BEST3 data collection from their treating clinician (see above). This letter will additionally explain that they may receive an invitation to participate in later stages of the study.

#### Aggregate data collection for BEST3 trial

Study entry will be defined as 14 days after the date the introductory letter is sent. At this point demographic and medication data will be extracted from the GP database for every patient entered into the study. This dataset will then be aggregated into sex and 10-year age groups (Table 4).

**Table 4:** Baseline and follow up data to be collected

Baseline data extract		12 month follow up extract	
Time point/period	Variable	Time point/period	Variable
Baseline	Sex	Baseline	Sex
	Age		Age
	Obesity records	Baseline and 12 months (where available)	Obesity records
	Smoking status		Smoking status
	Alcohol consumption		Alcohol consumption
Previous 12 months	PPI / H2RA prescriptions	Previous 12 months	PPI / H2RA prescriptions
	Other prescription medication: Aspirin, COX2i, antibiotics for *H. pylori* eradication		Other prescription medication: Aspirin, COX2i, antibiotics for H. Pylori eradication
	Heartburn and / or GERD related symptoms		Heartburn and / or GERD related symptoms
			Number of GP and practice nurse visits at home and at the practice
			Number of endoscopy or GI referrals
			Diagnosis of BE
			Diagnosis of EAC or pre-malignant conditions
			Diagnosis of benign oesophageal conditions
			Records on any upper GI specific procedures, e.g. endotherapies or oesophagectomies
			Anonymised endoscopy reports including pathology reports for BE and OC diagnosis; and benign conditions via a tick box (EoE, candida, inflammation, ulcer slough, squamous dysplasia, herpes, other)
			Type of referral: emergency via A&E, 2 week wait / urgent, routine and in or out patient (either form GP records or endoscopy report)
			Number of biopsies (from endoscopy reports)
			Anonymised letters from upper GI consultants

Follow-up data will be collected 12 months after study entry for all patients in each practice, irrespective of study arm and whether they had a Cytosponge™-TFF3 test. Where a new diagnosis of BE or EAC, and / or an endoscopy has been coded in the medical records, data will be extracted from endoscopy and pathology reports and entered into the aggregate data table manually (Table 4). Full baseline data will also be extracted.

#### Long term follow-up

When suitable anonymisation models become available, long term cancer registration and mortality data will be obtained from NHS Digital and the NHS Health and Social Care Information Centre (HSCIC) or equivalent. Data sent from HSCIC to QMUL will always be anonymised and aggregated by practice. Individually-consented participants in the trial may have their longer-term heath status followed up via data held by NHS Digital, HSCIC or its successor, the Office of National Statistics, Public Health England and other national databases.

### BEST3 intervention: Cytosponge™-TFF3 test

#### Patient invitation to BEST3 intervention

All participants in the intervention arm, who have not opted out of the study, will receive a second letter from their GP team inviting them to have the Cytosponge™-TFF3 test. This communication will include a standalone Cytosponge™ information leaflet (Additional file 1: Figure S1). This leaflet was developed based on findings from a qualitative study on the acceptability of the Cytosponge™ test [19] followed by several rounds of review by patient and public involvement (PPI) representatives. Once a participant has expressed interest by returning a reply slip or telephone call, the participant will receive a telephone call to further assess eligibility based on a short questionnaire. If eligible, an appointment will be arranged. The participant will then be sent a Cytosponge™ patient information sheet, consent form and appointment confirmation.

#### Cytosponge™ clinic procedure

Cytosponge™ clinics for participants from several practices will be held by either a CRN or local practice nurse with appropriate medical cover in place. Participants will be asked to refrain from eating and drinking for 4 h. Once consent has been obtained, the nurse will complete a questionnaire on demographic and clinical information and the previously validated GERD Impact Scale (GIS) with the participants [20] using case report forms (CRFs) in the BEST3 Database.

Patients will be asked to swallow the capsule with water. The capsule reaches the stomach while remaining attached to the string which is held onto by the patient or nurse (and which is affixed to a card preventing inadvertent swallowing of the entire string). In the stomach the capsule is left for up to 5 min where it dissolves allowing the sponge to expand to its full size. It is then withdrawn by the research nurse using the string, and as it does so collects cells from the lining of the oesophagus. The retrieved sphere is placed in preservative liquid. Linked anonymised samples will be sent directly from the practice to the pathology laboratory to be processed and analysed for TFF3 and H&E.

If a patient fails to swallow the capsule, they will be asked to try again. In the event of a Cytosponge™ detachment or an obvious bleed the research nurse will immediately inform the GP as the patient falls under their duty of care for medical assessment. Following medical assessment, the GP’s normal emergency procedures will be followed. The research nurse will telephone all participants who received the Cytoponge™ at 7 days post-procedure to assess safety and report adverse events.

#### *Storage and analysis of* Cytosponge™ *samples*

Cytosponge™ samples will be sent directly from GP sites to Cambridge University Hospitals NHS Foundation Trust Research Tissue Bank (CUHTB) to be processed into Formalin Fixed Paraffin Embedded (FFPE) blocks for TFF3 testing, and H&E analysis as described previously for the BEST2 trial [14].

#### Cytosponge™-TFF3 results

Patients will be informed about their Cytosponge™-TFF3 results by a standardised Cytosponge™ feedback letter from their GP within four to 6 weeks. Where the test is a low-confidence negative result, i.e. the sample fails in processing or is equivocal, the patient may be invited for a repeat test at a suitable location depending on local capacity. Other benign conditions of the oesophagus will be reported with the TFF3 result.

### Endoscopies

#### Invitation for endoscopies - intervention arm: TFF3-positive patients

Patients with a positive TFF3 test will be contacted by their GP to inform them of the result and offer an endoscopy examination to confirm the diagnosis. If this is agreed by the patient the GP informs the research nurse to arrange the procedure at the local endoscopy unit.

#### Invitation for research endoscopies - 10% of patients who do not require diagnostic endoscopy (all arms)

10% of the total number of study participants who have not had an endoscopy during the study will be invited for a research endoscopy at 12 months after entry into the study, including participants who have declined the original Cytosponge™-TFF3 intervention. Since BEST3 will be assessing the acceptability of endoscopy compared with Cytosponge™-TFF3, the intervention here will be “invitation to endoscopy”. Participants to be invited will be selected at random using the random selection function in practice databases. They will receive an invitation letter for a research endoscopy at their local hospital-based endoscopy clinic including an endoscopy-specific BEST3 Patient Information Sheet and a consent form.

#### Endoscopy procedures

Standard trans-oral endoscopy following BSG guidelines [21] for diagnosis of BE will be carried out. During the procedure the endoscopist will note the diagnostic endoscopic landmarks for BE using a standard protocol and in line with the Seattle protocol [22]:

- For all endoscopies where BE is found, biopsies will be collected (in all 4 quadrants) every 2 cm according to surveillance guidelines. In addition, endoscopically-suspicious areas will be targeted for biopsies.
- A further two biopsies from the gastro-oesophageal junction (GOJ) will be collected (below the z-line) for research purposes from both BE positive and patients with a TFF3 negative test.
- All biopsy samples will be processed and analysed by the local pathologist according to standard clinical practice including for benign conditions.
- Study participants will be informed about endoscopy findings in the usual way via a letter from the gastroenterologist copied to their GP.
- Endoscopic images will be requested of every GOJ and newly diagnosed BE to try to exclude misdiagnosed hiatus hernias and intestinal metaplasia (IM) at normal appearing GOJ.

#### Diagnosis of BE

Diagnosis of BE as the primary endpoint will be defined at three different levels of certainty:

- Diagnosis by the endoscopist or gastroenterologist following BSG guidelines
  - ∘ > 3 cm likely correct;
  - ∘ < 3 cm more suspect unless biopsy with IM;
- Confirmed by study pathologist or gastroenterologist
  - ∘ >C1 or >M3 +IM on biopsy;
- IM on biopsy
  - ∘ any length.

As a secondary endpoint we will also use a scoring system for BE according to severity (Table 5).

**Table 5:** Proposed BE scoring system

Score	BE severity
0	Pathology report not available
1	Intestinal metaplasia (IM) on biopsy and endoscopic findings not seen in categories below
2	C1 or C0 M3 + IM
3	C2 or more, C0 M4 or more +IM
4	C3 or more
5	Low grade dysplasia (LGD)
6	High grade dysplasia (HGD) or T1a cancer

TFF3 positive patients will also be asked to provide a saliva sample using the Oragene DNA kit at their endoscopy appointment. These samples will be stored for future genetic research.

### Acceptability measures- intervention group only

#### Patient acceptability measures

##### Baseline

Participants receiving the Cytosponge™-TFF3 test will be asked to complete a baseline questionnaire consisting of:

I. STAI-6, a short-form of the state scale of the Spielberger State-Trait Anxiety Inventory (STAI); this 6-item self-completed scale has been widely used to measure short-lived anxiety in relation to health experiences [23]. To aid the appointment process, and to ensure that the state being measured accurately reflects views about the procedure, the questionnaire will be provided to the patient prior to consent in the clinic waiting room area. If the patient does not go on to consent into the study, their questionnaire response will be disposed of securely.

Once consent has been obtained:

II. Lifestyle and family history questionnaire, covering education, history of smoking and alcohol, and any family history of heartburn, BE, oesophageal cancer and any other type of cancer.
III. Perceived risk of oesophageal cancer, using 2 items which have been widely used for other cancer risk assessments to assess perceived risk of developing EAC and perceived risk compared with a person of the same age (relative risk) [24];

##### 7–14 day follow-up

Seven to fourteen days post-study consultation, all participants receiving the Cytosponge™-TFF3 test will be either sent an email or text message with a link to an online questionnaire (or mailed a questionnaire as preferred). This questionnaire will consist of:

I. the Inventory to Assess Patient Satisfaction, used following flexible sigmoidoscopy screening, amended for the Cytosponge™-TFF3 test, and validated using face validation with 8 patients, who were either at high risk of BE or have had the Cytosponge™-TFF3 test; this has a 5 point ordinal scale with 22 items [25];
II. a visual analogue scale (VAS) in which 0 represents “Completely unacceptable” and 10 represents “Completely acceptable” [13];
III. Perceived risk of oesophageal cancer [24];
IV. STAI-6 [23].

If the follow-up questionnaire has not been returned after 2 weeks, a reminder will be sent to participants who have provided an e-mail address.

### Intervention acceptability for patients and health care professionals

#### Patients

Some participants from intervention practices will be interviewed to increase understanding of patient views on the Cytosponge™-TFF3 test and its use in the primary care setting. Up to 30 patients across a range of ages and including both men and women, will be interviewed within six to 8 weeks of their trial consultation. Patients will be interviewed in their own home or a place of their choosing. They will provide written consent prior to interviews commencing.

Data will be audio-recorded and transcribed professionally. It will remain confidential and will not be shared with their GP. Data will be analysed using Thematic Analysis [26], supported by NVivo. We expect the following themes to be explored in the analysis: patient views of acceptability of Cytosponge™-TFF3 test use in primary care, and patient understandings and perceptions of ‘risk’ in relation to their symptoms. As qualitative analysis is an inductive and iterative process, further themes will evolve throughout the analytical process.

#### Healthcare professionals

##### GPs

Semi-structured interviews with up to 20 GPs from intervention practices will be undertaken to identify and gain an understanding of the facilitators and constraints influencing use of the Cytosponge™ in primary care routine clinical practice. GPs will be recruited purposively to sample as widely as possible (region, gender, age, trainer status, rural / urban location). GPs can choose to be interviewed face-to-face or by telephone. Those who are interviewed by telephone will be asked to provide verbal consent at the beginning of the interview and also complete a written consent form to be returned by post. Data collection, transcription and analysis will be undertaken in a similar way to the patient interviews.

##### Research nurses

All research nurses involved in delivering the intervention will be asked to complete a short on-line questionnaire at the beginning and end of their involvement in the study. This will focus on issues around their training, patient recruitment to their clinics, and delivering the intervention. We will also undertake semi-structured interviews with up to 20 research nurses from intervention practices, to identify and gain an understanding of the facilitators and constraints influencing use of the Cytosponge™-TFF3 test in primary care routine clinical practice. Research nurses will be recruited to sample as widely as possible (region, gender, age, trainer status, rural / urban location), for a telephone interview. The methods of data collection, transcription and analysis will be identical to the GP interviews. In addition, we will undertake descriptive analyses of the questionnaire data, and use both datasets in a mixed methods analysis to look for overarching themes.

### Involvement of public and patient involvement representatives

The BEST3 study design and key documents (introductory letter, Cytosponge™-TFF3 test leaflet, and PIS) were discussed with a group of seven PPI representatives based at Cambridge University Hospitals NHS Foundation Trust. This group reviewed all patient facing materials. Their advice will be used to further guide any changes to the design of the study and study materials. There are two active lay members of the BEST3 trial steering committee (TSC). As part of the TSC, they are involved in the remainder of the trial, including analysis of results and dissemination of findings.

### Statistics and data analysis

#### Sample size calculations

The sample size calculation is based on the following assumptions:

i. BE prevalence in individuals eligible for the study is 4–5%;
ii. 10% of patients in the usual care arm will be referred to endoscopy for clinical reasons (after excluding urgent referral);
iii. The prevalence of BE in patients referred to endoscopy in the usual care arm is 6%;
iv. Uptake of the Cytosponge™ test is currently expected to be 50%;
v. Cytosponge™-TFF3 sensitivity is 85%  [14];
vi. Endoscopy sensitivity is 100%.

The assumed 4% prevalence of BE in individuals eligible for the study was based on the BEST1 trial, which reported 3% in a population, but included patients with less severe GORD compared with the BEST3 trial [13]. Since only 50% of patients in the Cytosponge™ arm are predicted to have the Cytosponge™ test and patients who do not take up the offer of the test will have the same management as if they were in the usual care arm we only expect 2.0% of patients in the intervention arm to be diagnosed with BE. Furthermore, we expect 0.6% of patients in the usual care arm to be diagnosed with BE. For a 90% power (not allowing for clustering) comparing 0.6% with 2.0%, we would need 3028 individuals (1514 in each arm).

To account for the fact that individuals within a cluster, here a GP practice, might be more similar to each other than to individuals in other clusters, the number of participants required for an individually randomized trial has to be increased. The sample size calculations were adjusted for cluster randomization using the variance inflation factor (VIF) [27]. We allowed for variation in practice size and adjusted the sample size calculations for three groups of practices recruiting either 50–60, 61–74 and 75–100 patients. Allowing for intra-cluster correlation of 0.025, an overall coefficient of variation of 0.2, and mean cluster sizes of 55, 68 and 88 the VIF was estimated as 2.4, 2.7 and 3.3 respectively. Multiplying the VIF with the sample size estimated for individual randomization for each stratum, we would need a total of 8488^1^ patients. If we are recruiting three groups of 40 practices with 50–60, 61–74, or 75–100 patients each, then 120 practices will result in approximately 90% power. Assuming 50% uptake, this would result in 2122 patients having the Cytosponge™ test. If the uptake is lower, the overall number of patients entered into BEST3 will be increased so that still a total of 2122 patients will have the Cytosponge test.

### Statistical analysis

#### Primary endpoint

##### Null hypothesis

The BE detection rate at 12 months (excluding any found on random exit endoscopies) is the same in the intervention (Cytosponge™-TFF3 test) arm and the control (usual care) arm.

##### Alternative hypothesis

The BE detection rate at 12 months is not the same in the intervention (Cytosponge™-TFF3 test) arm compared with the control (usual care) arm.

To determine whether the invitation to the Cytosponge™-TFF3 test leads to an increase in the number of patients diagnosed with BE compared with the usual clinical care pathway we will compare BE diagnosis between intervention and the control arms in all patients entered into the study. We shall compare the proportions of BE between the two groups at 12 months after study entry using a generalised estimating equation (GEE) with BE diagnosis as the binary outcome comparing the two arms as fixed effects with adjustment for age, gender, BMI, and length and dose of acid suppressive treatment together with cluster as a random effect. We will use both the assumed intra-class correlation (0.025) and the estimated intra-class correlation. Statistical significance will be based on a two-sided test with alpha equal to 5%.

**Secondary endpoints (all to be estimated together with (nominal) 95% Confidence Intervals):**

1. To estimate the diagnostic accuracy of the Cytosponge™ in primary care the sensitivity will be calculated as the proportion of TFF3 positive tests in patients with endoscopy confirmed BE (gold standard). The specificity will be calculated as the proportion of TFF3 negative tests in endoscopy confirmed BE negative patients amongst the 10% participants randomly invited to endoscopy from TFF3 negative patients.
2. To confirm the prevalence and incidence of BE and cancer stage diagnosis in the intervention and control arms for 10 years: Long term follow up data will allow Cox proportional hazard model analysis to compare oesophageal cancer and death rates.
3. The acceptability of both Cytosponge™ and endoscopy tests will be assessed as the proportion of patients willing to have the test among patients offered the test. 95% confidence intervals (Clopper-Pearson) will be used.

### Economic evaluation

#### Primary endpoint

To gain an in-depth understanding of the health economics of the Cytosponge™-TFF3 test we will undertake a detailed analysis of the cost and cost-effectiveness of the Cytosponge™-TFF3 test versus usual care from the perspective of the NHS and personal social services. For the cost analysis, cost components will include costs of diagnosis using the Cytosponge™-TFF3 test, endoscopies and biopsies, endotherapy, oesophagectomy, and follow-up in primary and secondary care. Volume of resource use data will be collected from practice records up until 12 months after study entry. Unit costs will be taken from published sources such as NHS Reference Costs, the British National Formulary, and the Personal and Social Services Research Unit (PSSRU). We will calculate mean (SD) and median (IQR) costs for both study arms for each cost component and all components combined.

Cost-effectiveness will be measured in terms of the incremental cost per QALY gained. We will adapt a previously-developed decision analytic model which compared the cost-effectiveness of three strategies: Cytosponge™ screening with confirmatory endoscopy, endoscopy alone, and no screening, for 50 year-old men with GERD. The parameters for prevalence of BE, sensitivity and specificity of screening, uptake of the Cytosponge-TFF3 test, costs and utilities will be updated to reflect the BEST3 data. For these and all other parameters, including disease progression and management strategies, we will search the literature for updated evidence. We will extend the modelled population to also include women over 60 years, obtaining gender/age-specific parameters from BEST3 or searches of the literature where necessary. We will also undertake value-of-information (VoI) analyses, based on the notion that investing in further research on probabilities of events, HRQL and costs will reduce decision uncertainty about the cost-effectiveness of the Cytosponge-TFF3 test. This will include both the expected value of perfect information (EVPI) and the expected value of partial perfect information (EVPPI). The latter focusing on individual model parameters or groups of parameters.

As well as looking at total costs, costs will be disaggregated by sector since this may affect implementation of the Cytosponge™-TFF3 test. Using epidemiological data on the national incidence of reflux predominant symptoms plus cost data from the present study we will also undertake a budget impact study to calculate what the total cost would be to the NHS if the Cytosponge™-TFF3 test was rolled out nationally.

### Pilot phase and 6-month milestone review

The study design described here is the original design submitted in the first version of the protocol for Health Research Authority approval including ethical review. This protocol was used for the pilot phase of BEST3, which consisted of the first 6 practices, to evaluate the feasibility of this design. The study design was then amended based on the findings from the these practices (uptake = ~ 30%; male: female ratio = 46:54%) (Table 6). In brief, patients will receive one reminder about their invitation to make a Cytosponge™-TFF3 test appointment. Furthermore, we may adopt a shorter follow up period for practices recruited later in the trial using a simulation tool to ensure parity across datasets. This will allow timely completion of the trial. Overall, the pilot phase showed that the study design was logistically sound and that the full study could go ahead.

**Table 6:** Amendments to the study protocol post pilot phase

Pilot study findings	Amendments to study protocol
Female to male ratio slightly higher than 50:50 overall	If the proportion of females to males consistently exceeds 55:45 within the overall cohort, the study team may institute a 50:50 split for females: males in line with known BE prevalence, at the discretion of the Trial Statistician.
Cytosponge™ appointment uptake < 50%	Patients will receive a total of one reminder in the form of a letter, phone call or text message.
Time required for practice and patient recruitment might take longer for each practice than anticipated	Practices recruited in the latter stages of the trial may adopt a 6 month follow-up period to allow timely completion of study activities with a simulation tool used to ensure parity across the datasets.

Due to the continuing low uptake of less than 30% further amendments were made after the 6-month milestone review (Table 7).

**Table 7:** Amendments to the study protocol post 6 months milestone review

Milestone review findings	Amendments to study protocol	Details
Cytosponge appointment uptake 27%: Substantial impact on sample size as number of practices would have to be increased to ~ 200	1) Sample size amended - Using uptake = 27% - including patients with false negative Cytosponge test diagnosed with BE during 12-month follow-up: (1–0.85)*0.6% 2) Additional individual randomisation arm added to reduce the sample size	- Individual randomisation sample size (without adjusting for cluster randomisation): 6764 - Variance inflation factor (VIF) for confirmed and projected sample sizes are 3.72 and 4.5, respectively. 1 individually randomised participant would therefore be equivalent to 3.72 or 4.5 cluster randomised participants - Practices already commenced set up on the cluster randomisation design allowed to continue to randomise in a cluster fashion - Sample size will be adjusted depending on number of patients in cluster randomised group
Due to small number of smaller practices, stratification by both area and practice size has resulted in imbalances in arm allocations for some areas	Stratification by practice size will not be taken into account during randomisation but in the analysis instead	- To simplify randomisation and avoid any further imbalances for remaining cluster randomisation practices - Analysis: the primary analysis will be a stratified test of proportions taking into account the variation inflation within each stratum.

Firstly, to obtain sufficient power without greatly expanding the number of participants and practices required, we plan the addition of an individually randomised group to the current design. Practices already setup or trained to take part in the cluster-randomised arm will be permitted to continue with this randomisation method. Newly engaged practices will randomise using individual randomisation methods. The sample size calculations have also been updated to include patients in the intervention arm with a false-negative Cytosponge™-TFF3 test as having the same chance of a BE diagnosis via routine endoscopy as in the control arm (10%). This adjustment and the updated uptake of the Cytosponge™-TFF3 test allowed us to estimate that about 1.4% patients in the intervention arm will be diagnosed with BE within 1 year of follow-up. Requiring 90% power and a significance level of 0.05% gives am updated sample size of 6764 individually randomised patients. The VIF for practices randomised up to now is 3.72, whereas we are using a VIF of 4.5 for practices which have not been randomised yet as we do not know the number of recruited patients yet. One individually randomised participant would therefore be equivalent to 3.72 or 4.5 cluster randomised participants. Based on confirmed and projected numbers of participants in cluster-randomised practices, we anticipate 11,816 patients from 100 practices contributing the equivalent of 2924 individually randomised patients. That would leave 3840 to be recruited from practices employing individual randomisation and a total sample size of 15,656 participants overall.

Secondly, due to small numbers of smaller practices, stratification by both area and practice size has resulted in imbalances in arm allocations for some areas. Stratification by practice size will not be taken into account during randomisation anymore, but in the analysis instead.

These changes will ensure that the sample size will be sufficiently large to detect differences in BE diagnosis between the two arms and that the study will be completed in the time frame required.

## Discussion

The BEST3 trial is a multi-site cluster-randomised controlled trial to evaluate the ability of the Cytosponge™-TFF3 test to identify BE among patients with gastro-oesophageal reflux predominant symptoms not meeting guidelines for referral. This study design is set within the MRC framework for the design and evaluation of complex interventions [17]. The reasons for choosing this design over other designs are:

1. We are not proposing this as a screening test per se, but as a triage test in people being treated for GERD symptoms with an acid suppressant drug.
2. We are not looking at death from EAC because that would require too big a study and it was not considered feasible when discussed with experts.
3. We considered directly comparing the Cytosponge™-TFF3 test to endoscopy, but have rejected that option because we do not envisage endoscopy ever becoming the usual care for the majority of people with GERD. Apart from anything else, there are simply not enough endoscopists. Additionally, BEST2 has established that the sensitivity to BE of the Cytosponge with TFF3 staining is very high.
4. The key question is to know how the use of the Cytosponge™-TFF3 test would work in practice. Hence, we need a pragmatic clinical trial and the most appropriate control arm is “usual care”.
5. We considered individual randomisation in the original trial design, but rejected it because we were planning to offer the Cytosponge prospectively to patients who consulted about reflux. As some patients would have had more than one consultation during the recruitment period there was a risk that they would get randomised more than once. Furthermore, it might have been difficult for GPs to remember which arm a patient would have been in, and how to advice them. As we are now recruiting prevalent patients, these issues would not occur. However, cluster randomisation was initially chosen as it makes the day to day management of the trial easier for general practice teams.
6. There is also a “mechanistic” element to the trial. People are also interested in what might be the features of individuals who have a false negative Cytosponge™-TFF3 test and what other benign diseases (of the oesophagus or the stomach) would be diagnosed or missed by offering the Cytosponge™-TFF3 test to patients. Hence, we propose offering endoscopy for research purposes to a sample (10%) of patients.
7. Analysis of a CPRD cohort has shown that patients with at least 6 months of prescriptions for an acid suppressant are the most suitable for this trial, as they had the highest conversion rate to BE (manuscript in preparation). Eligible patients will therefore be identified and invited based on a GP database search.
8. Informing patients in the usual care arm about this increased risk and a new diagnostic tool for the diagnosis of BE, but not giving them the opportunity of having this test, could result in increased patient worry.
9. A qualitative study carried out to aid in the design on the BEST3 trial showed that the majority of participants were not aware of the increased risk of BE and oesophageal cancer [19]. Some participants felt that telling patients who are being invited to have the Cytosponge™-TFF3 test about the link between GERD, BE and cancer would scare them. Furthermore, usual care arm patients might be requesting the Cytosponge™-TFF3 test in practices where it is not available or endoscopies creating additional burden for endoscopy services. Therefore, patients in both arms will first only be informed about the BEST3 anonymous data collection, but not told about the Cytosponge™-TFF3 test. Patients in the BEST3 intervention arm will then be separately invited to the Cytosponge™-TFF3 test. The PPI representatives based at Cambridge University Hospitals felt this study design was appropriate.

With the incidence of EAC having increased dramatically over the last 30 to 40 years and it being the highest in the UK worldwide, this test has the potential to reduce the number of late diagnoses and therefore increase 5-year survival significantly. The BEST3 trial is a well powered pragmatic trial testing the use of the Cytosponge-TFF3 test in the same population that we envisage it being used in clinical practice and will be reasonably short lasting only 3 years. The data generated from this trial will enable NICE and other clinical bodies to decide whether this test is suitable for clinical use.

## Additional file

Additional file 1: **Figure S1.** Cytosponge™ information leaflet. This leaflet was designed based on the findings from a qualitative study investigating the acceptability of and information preferences on this test among patients with GERD. A combination of text and visual illustrations was used to provide easily accessible information on the Cytosponge-TFF3 procedure, the link between GERD, BE and EAC, and BEST3. (PDF 669 kb).

## Abbreviations

BE: Barrett’s oEsophagus
BEST3: Barrett’s oESophagus Trial 3
CPRD: Clinical Practice Research Datalink
CRF: Case report form
CRN: Clinical research network
CUHTB: Cambridge University Hospitals NHS Foundation Trust Research Tissue Bank
EAC: OEsophageal adenocarcinoma
EMIS: Egton Medical Information Systems
EVPI: Expected value of perfect information
EVPPI: Expected value of partial perfect information
FFPE: Formalin Fixed Paraffin Embedded
GEE: Generalised estimating equation
GERD: Gastro-oesophageal reflux disease
GIS: GERD Impact Scale
GP: General Practice
H2RAs: H2 receptor antagonists
HRQL: Health related quality of life
ICER: Incremental cost effectiveness ratio
IQR: Interquartile range
NSAIDs: Non-steroidal anti-inflammatory drugs
PPIs: Proton pump inhibitors
PSSRU: Personal and Social Services Research Unit
QALY: Quality adjusted life year
SD: Standard deviation
STAI: Spielberger State-Trait Anxiety Inventory
TFF3: Trefoil Factor 3
TSC: Trial steering committee
VAS: Visual analogue scale
VIF: Variance inflation factor
VoI: Value-of-information

## Footnotes

1 The sample size in the protocol originally submitted for ethics review was 8988 due to an error, which has been corrected here.
