# Supplementary material for: Barrett’s oESophagus trial 3 (BEST3): study protocol for a randomised controlled trial comparing the Cytosponge-TFF3 test with usual care to facilitate the diagnosis of oesophageal pre-cancer in primary care patients with chronic acid reflux
Source: BMC Cancer. 2018 Aug 3;18:784. doi: 10.1186/s12885-018-4664-3 (PMC6091067; doi:10.1186/s12885-018-4664-3)
Supplement: Supplementary file 1 — Figure S1. Cytosponge™ information leaflet. This leaflet was designed based on the findings from a qualitative study investigating the acceptability of and information preferences on this test among patients with GERD. A combination of text and visual illustrations was used to provide easily accessible information on the Cytosponge-TFF3 procedure, the link between GERD, BE and EAC, and BEST3. (PDF 669 kb). [file 12885_2018_4664_MOESM1_ESM.pdf]

Frequently asked questions

How big is the Cytosponge capsule?

About the same size as a paracetamol capsule.

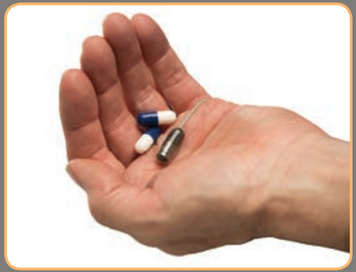

How big is the Cytosponge when not compressed?

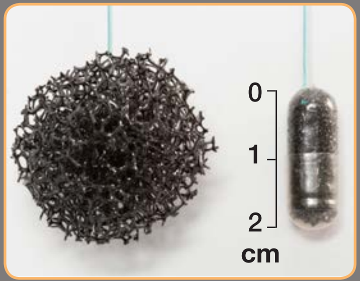

It is a soft sponge about the size of a 50 pence coin.

Will the procedure make me gag?

The water makes it easier to swallow the capsule and string. You may gag when the nurse pulls the sponge out, but it will only take a second or two.

Can I get something to numb my throat?

Most people prefer not to have a numbing spray but this is available if you would like to have it.

What happens if the string breaks?

This is extremely unlikely. However, if it does, the sponge will stay in the stomach, which is harmless. We will arrange for you to have an endoscopy the same day to remove the sponge.

What are the side effects?

You might have a sore throat for a few hours after the procedure, which can be soothed with a sweet or lozenge.

thebmjawards

Winner of The BMJ 2016 Gastroenterology Team Award

Winner of the 2011 NHS Innovation Challenge Prize

UNIVERSITY OF CAMBRIDGE School of Clinical Medicine

MRC Cancer Unit

REC 16-EE-0546, IRAS: 210292, BEST3 Cytosponge Trifold leaflet v1.0’

Invitation for a Cytosponge™ test as part of the BEST3 Trial

for people with heartburn, reflux and/or indigestion

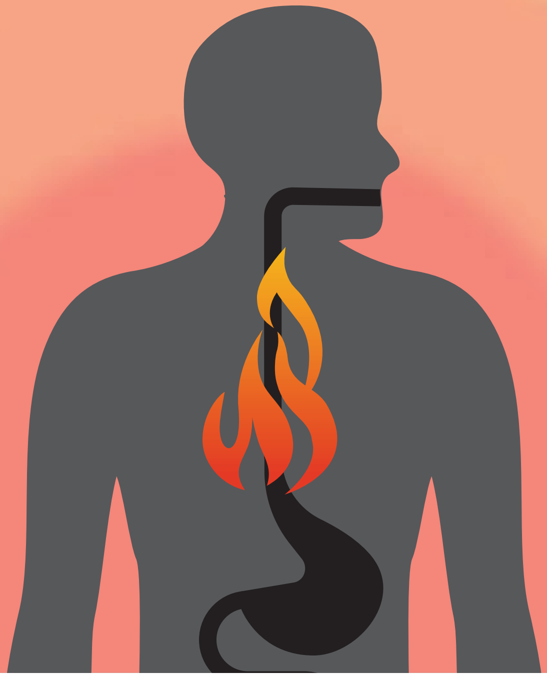

CANCER RESEARCH UK

Why are we offering the Cytosponge™ test?

The Cytosponge is a capsule on a string, which expands into a small sponge in the stomach. The Cytosponge test can find conditions of the oesophagus that are more common among people who have heartburn, reflux and/or indigestion.

When stomach acid or bile irritates your stomach, upper bowel or oesophagus, this is known as indigestion. Acid reflux is when stomach acid flows up the oesophagus. This can cause pain in the chest, neck or throat, also known as heartburn.

The Cytosponge has been tested in over 3000 patients. It is quick, safe and accurate.

Acid reflux and cancer

About 3-6 people out of 100 with regular heartburn, reflux or indigestion will develop a condition called Barrett's oesophagus or Barrett's.

People with Barrett's oesophagus have a slightly higher chance of developing oesophageal cancer.

Regular monitoring can help to prevent cancers from developing. It can also help to find cancer when it is easier to treat and cure.

How does the Cytosponge test work?

1) You will put a capsule in your mouth and take a large sip of water.

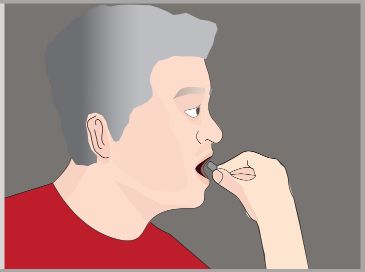

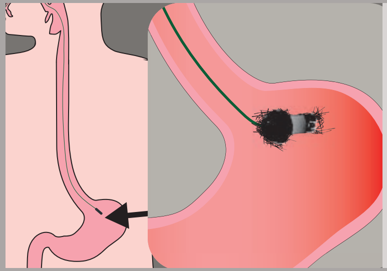

2) It will take up to 5 minutes for the capsule to dissolve, releasing the sponge in your stomach.

3) The nurse will pull up the sponge by the string. This will only take 1 or 2 seconds.

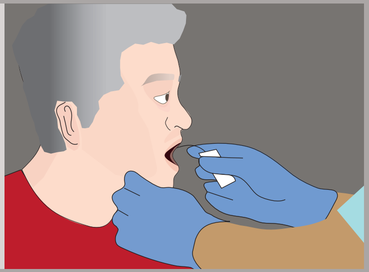

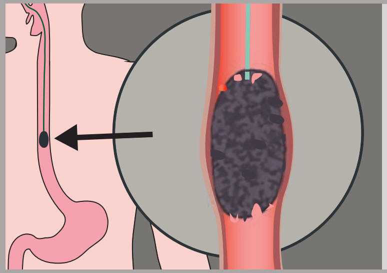

4) The sponge collect cells as it passes through your food pipe.

5) Experts will look at the sample to see if you have signs of Barrett's oesophagus.

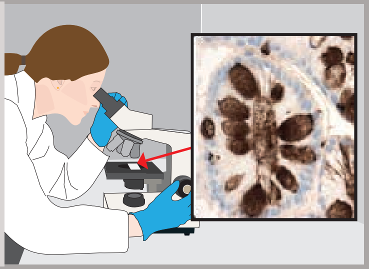

The BEST3 Trial

Our research has found that the Cytosponge test is accurate at detecting Barrett's oesophagus.

The BEST3 trial will test if the Cytosponge is a good way to find Barrett's when it is used in GP surgeries.

Why have I been invited?

Your GP thinks this a good study and has decided to enrol patients in it. Your GP records show you are aged 50 or older and have been prescribed medication used to treat heartburn, acid reflux or indigestion.

What will I need to do?

You will have one Cytosponge test at your GP surgery. We will then let you know the results within 4-6 weeks by post

If your results are positive for Barrett's it does not mean you have cancer. However, you will be monitored every few years for early signs of cancer.

How can I take part?

If you are interested to find out more please complete the enclosed reply slip and return it in the freepost envelope. Alternatively you can call your GP.

Find out more on
http://www.best3trial.org/
